# Supplementary material for: Expression of GEX1 Orthologs of Brassica rapa and Oryza sativa Rescued the Nuclear Fusion Defect of the Arabidopsis GEX1 Mutant
Source: Plants (Basel). 2022 Jul 8;11(14):1808. doi: 10.3390/plants11141808 (PMC9324357; doi:10.3390/plants11141808)
Supplement: Supplementary file 1 [file plants-11-01808-s001.zip › plants-1771949-supplementary.pdf]

```
                                                                                                                          CRD
AtGEX1 MDRFSRKCLLFLLLIILLDSPL--TCHSWGWFSSSSSSAEDPYSSS----FSRSRKSNPDFSMEVFSDQKAVQVLE---NKLVGLTSCWQNAYSYLLAGCKETIATEEKR 101
BrGEX1 MDHFKRRYLLFL-LITLIDSPI--TCHSWGWFSSSGENTN----PS----SSRSIKSNTEFSIEVFSDKKAVQVLEDAKNKLAGPNSCWQNAYGYLLSGCKGMVATEEQR 99
OsGEX1 MRRHAGTIILLLILACLWLCPGRSSGFSWNIFSSSSSSPPTAGESRAAPMLELDGAVADFLMDGADDPRAVKLLENARSKLAGPSNCWQEAYRRLFASCGDIMADKEMQ 110

AtGEX1 KRFAWYLSDCFIKDSGRPAFPTCKDESVMMSCLKKLDDHEHKIYLDFLLETNTICQQLQSNAFKNEIERLVNELKNTAQYTEDKLDILESKSDALIQTSSMIHDSLGSLD 211
BrGEX1 KRFAWHLSDCFQKESGRPDFPTCNDKQTMMSCLKKLDDHEHKIYLEFMLETNTICQQLQSHALKNEIERLVNDLKRSAQNTEEKLDILESKSDDILQGTSKIHESVGLID 209
OsGEX1 SRLAWHLSSCFQEDSGRPPFPRCGEVSDMVHCRKRLGVSEDQVFLEFFLETNTLCHQLQAEAFKHNTERLVNDLTRTSKSAEEKLEVIEERSDQIIQESRKVQETISSIE 220

AtGEX1 VRVQNVASVTNTLETSVSGLSQQTVEISQEQKNIAESQLALRDGQVKMKETLKDGMDMFLDAYTNIQEGVDKLKSDTEQIEVEISVLGNNLSTKMIDLQSTTDDIGTKTR 321
BrGEX1 VVVNNVAHKTNTIGTQMSGLSQQTRDIYQEQKSITESQLALKEGQEKMGEAMKVGMEMFNDSVTDVKDGVDRLKNDTKQIGGKINQLGEKMSEKMTALENQTSIIGTMTN 319
OsGEX1 MQADHLAEASKNVGDQIDDVLAHSKAIFEQSKEIADSQAKLREGQTEMRETIDAGMTHIQESYESLGNGMDKLKEEAVDIQREIKTVGDSMSTKMQDLQSTANDIGSVAG 330

AtGEX1 SSLDKQQKLLDGQTVALDGIQFLTRFQSEALQESRNTLQRLKEFSQEQQEDLAKRQEKLQEVHDHLFENSKSMLEAQVAFEAKQANMFVALDKLFALHNAMLLESRVIKA 431
BrGEX1 STLDNQQKLLEGQSVAIDSIQSLNQFQSEALQESRSTLQRFAEFSQEQQEDLAKRQEQLQQVHDHLFENSKSMLAAQEAFEAKQASMFVALDKLFALHNAMLLESRVIKA 429
OsGEX1 KSLENQMQLLDGQSKAMDGLNNLYSFQAQALEESRETVQKLAQFGQRQQEELLSRQEEIRQAHEHLIHNSHSILEAQEEFRAKQANIFAALDKLYILHNAILAESRFIKA 440

AtGEX1 FVIYFLSIFVIYMFTSTKQTYIIRPRLYIGLCVTLALEVASLRYVN-DTERQAWMIN---LIRSLFALLASAQLLHAALSYRDYEVLNHQILLRLVDKVNDMQSKK---- 533
BrGEX1 FFIYFLSIFVIYMFTSTKQTYTIRPRLYIGLCVTLALEVASLRYVN-DEEHRAWVIN---VLRSLFAVLASAQLLHSAFTYRDYEVLNHNILLRLVDKVNSMQSKR---- 531
OsGEX1 FFFYCCIVFLIYMLTSAKQTFSIRGHLYFGLCITLLLEIGLIKLGADDIDKQFWVISKVFLVRSVFLALATVQMLHAIFTFRDYELLNHHLLQTLVEKVRALEETAAAGE 550

AtGEX1 -ELSYDE---DTESEVDWTSWVDTDLTDDDDNLADPDYKIPLLIKDNPVTT-------------------SSLTRRLYNFRPR------ 593
BrGEX1 -EILWDE---DTDSEVDWNSWIDTDITDDDDSLGDPDYRIPEQIKDNVGFT-------------------SSMTKRLYNLRPR------ 591
OsGEX1 KMLPYGGGGAESERSLMDYSWVFDELADEVDSNADPSYALPGDEQRQVAVVAPRRRHCASPEEVVGENSITTSAGRRYNLRPRSSYRQT 639
```

% identity

	BrGEX1	OsGEX1
AtGEX1	73.81	43.84
BrGEX1		42.13

Figure S1. Sequence alignment of AtGEX1 (At5g55490), BrGEX1(XP_009120059) and OsGEX1 (XP_015611623). Alignment was performed using Clustal Omega (https://www.ebi.ac.uk/Tools/msa/clustalo/). Amino acid residues that do not match with AtGEX1 are highlighted in pink. CRD is shown as a box. Percent identities between each sequence are shown below the alignment

Table S1. PCR Primers Used for Plasmid Construction.

Name	sequence（5'-3'）
BrGEX1F1	GCCGCCCCCTTCACCATGGATCATTTTAAAAGAAG
BrGEX1R1	GGCGCGCCCACCCTTTTATCGTGGACGGAGATTGT
OsGEX1F2	GCCGCCCCCTTCACCATGAGGAGACACGCAG
OsGEX1R2	GGCGCGCCCACCCTTTCACGTCTGCCTGTAACTACTCCG

Table S2. PCR Primers Used for Quantitative Real-Time PCR.

Gene name	Direction	Sequence (5'-3')
AtGEX1 (At5g55490)	Forward	GGCTTTACATTGGTTTGTGCG
	Reverse	ACTTCATAGTCCCTGTATGACAAAG
UBQ10 (At4g05320)	Forward	GGCCTTGTATAATCCCTGATGAATAAG
	Reverse	AAAGAGATAACAGGAACGGAAACATAGT
BrGEX1	Forward	TCTGTCACTGACGTCAAGGTAGG
	Reverse	TCTCCAACGCAGTCATCTTCTC
OsGEX1	Forward	GAGAGCCTTGGCAATGGGAT
	Reverse	ATGGCCTTGCTCTGTCCATC
